# Results of Infrapopliteal Endovascular Procedures Performed in Diabetic Patients with Critical Limb Ischemia and Tissue Loss from the Perspective of an Angiosome-Oriented Revascularization Strategy

**DOI:** 10.1155/2014/270539

**Published:** 2014-01-06

**Authors:** Francisco Acín, César Varela, Ignacio López de Maturana, Joaquín de Haro, Silvia Bleda, Javier Rodriguez-Padilla

**Affiliations:** Department of Vascular Surgery, Hospital Universitario de Getafe, Carretera de. Toledo Km 12.5, Getafe, 28905 Madrid, Spain

## Abstract

Our aim was to describe our experience with infrapopliteal endovascular procedures performed in diabetic patients with ischemic ulcers and critical ischemia (CLI). A retrospective study of 101 procedures was performed. Our cohort was divided into groups according to the number of tibial vessels attempted and the number of patent tibial vessels achieved to the foot. An angiosome anatomical classification of ulcers were used to describe the local perfusion obtained after revascularization. Ischemic ulcer healing and limb salvage rates were measured. Ischemic ulcer healing at 12 months and limb salvage at 24 months was similar between a single revascularization and multiple revascularization attempts. The group in whom none patent tibial vessel to the foot was obtained presented lower healing and limb salvage rates. No differences were observed between obtaining a single patent tibial vessel versus more than one tibial vessel. Indirect revascularization of the ulcer through arterial-arterial connections provided similar results than those obtained after direct revascularization via its specific angiosome tibial artery. Our results suggest that, in CLI diabetic patients with ischemic ulcers that undergo infrapopliteal endovascular procedures, better results are expected if at least one patent vessel is obtained and flow is restored to the local ischemic area of the foot.

## 1. Introduction

Critical limb ischemia (CLI) mainly affects elderly patients with important comorbidities and significant diffuse multilevel vascular lesions [1, 2]. These patients are frequently diabetics with neuroischemic limb ulcers, gangrene, and foot sepsis. This specific group is prone to develop an aggressive form of the disease, with more tibial affectation and microcirculatory impairment [3, 4]. The risk of limb loss is higher among diabetics and in patients with ischemic ulcers [1, 2]. In the absence of a successful revascularization, major amputation and mortality rates of CLI patients are substantial. In this context, obtaining at least one patent tibial artery to the foot is usually needed to achieve a sufficient amount of blood flow to cover the healing process requirements and ensure limb salvage [5–9]. Therefore, infrainguinal revascularization procedures are frequently performed, especially on tibial vessels. According to several studies, tibial endovascular techniques could provide similar clinical outcomes as distal vein bypass surgery with a lower rate of procedure-related complications [10–12]. In many centres, these interventions have been implemented as first line of treatment for CLI as it seems more suitable for this frail group of patients because of its lower perioperative adverse event rates.

In tibial vein bypass planning, it has been generally accepted that the decision of which tibial outflow vessel should be treated must be taken according to angiographic considerations of the target distal vessel quality, since better patency rates are expected if less diseased runoff artery is used for distal anastomosis. However, the endovascular approach offers the possibility of treating more than one tibial vessel. In addition, data shown in several recent reports suggest that improved clinical outcomes could be achieved when blood flow is directed to the local ischemic area of the foot through its specific source tibial artery using and angiosome anatomical model [7–9, 13]. This revascularization strategy is usually hampered by the seriously affected vascular anatomy of CLI patients. In fact, in a large number of patients, the restoration of blood flow to the ulcerated area could only be achieved through distal collateral vessels of the foot like distal peroneal branches or pedal arch [9].

Our aim is to analyze the clinical and hemodynamic results of infrapopliteal endovascular procedures applied to CLI diabetic patients with ischemic ulcers according to the number of tibial vessels attempted for revascularization, the number of tibial arteries finally achieved to the foot and the local perfusion of the ischemic ulcer obtained after revascularization in order to describe the usefulness of these recent techniques in new reperfusion strategies.

## 2. Patients and Methods

We carried out a retrospective study of consecutive primary infrapopliteal endoluminal techniques performed at our Vascular Surgery Department for CLI and ischemic ulcers in diabetic patients over a ten-year period (January 1999–December 2009). All patients with end-stage renal disease were excluded from the analysis since this specific condition is associated with worst outcomes due to the extensive arterial calcification found in these subjects. During this period, endovascular procedures were progressively implemented as the first treatment option for CLI whenever technically feasible. The clinical diagnosis of CLI was confirmed by objective documentation of severe hemodynamic compromise according to TASC (TransAtlantic InterSociety Concensus) criteria [1]. Absence of pedal pulses and documentation of severely compromised hemodynamics using ankle-brachial index (ABI) were sought. However, toe pressure was not routinely measured on those with noncompressible ABI. The indication for revascularization was based on color duplex scanning and angiography.

Demographic details, atherosclerosis risk factors, basal and postoperative hemodynamic data, and TASC classification for the worse lesion treated were entered in our database. Femoropopliteal lesions were classified using the TASC-II criteria [1] and infrapopliteal lesions were classified according the TASC criteria published in 2000 [2]. Patients were defined as being hypertensive if they had been diagnosed as such (systolic blood pressure >140 mmHg and/or diastolic blood pressure >90 mmHg) and/or had been on antihypertensive treatment for at least one year. Patients with plasma total cholesterol >250 mg/dL, LDL-cholesterol >160 mg/dL or triglycerides >200 mg/dL, or on lipid-lowering treatment were defined as dyslipidemics. Patients were considered to suffer from diabetes mellitus if they had baseline blood glucose levels >120 g/dL or required treatment with hypoglycaemics. Current and past smokers were considered to have history of smoking. Patients with previously diagnosed of acute myocardial infarction and/or angina pectoris and those who required coronary revascularization where considered to suffer ischemic heart disease. Patients with previous history of ischemic stroke or transient ischemic attack were recorded. Those subjects who had been diagnosed with chronic bronchitis or emphysema were considered to suffer chronic obstructive pulmonary disease.

Our cohort was divided in groups according to the number of tibial vessels attempted for treatment, the number of patent tibial vessels finally achieved to the foot and the local perfusion of the ischemic ulcer obtained after revascularization. Thus, the infrapopliteal interventions were classified as “single revascularization” if only one tibial vessel was treated and “multiple revascularization” if more than one infrapopliteal vessel was attempted for revascularization.

We considered that a patent tibial vessel was finally achieved if lesion recanalization was successful and morphological result of the procedure on target lesion was optimal (without dissections and/or without residual stenosis >30%). Endovascular treatment of the peroneal artery also required at least one patent distal peroneal branch to the foot to consider the artery successfully revascularizated. Those procedures in which none patent tibial arteries were obtained were classified as “runoff 0 group.” Interventions in which a single outflow vessel to the foot was obtained with an optimal morphological result were classified as “runoff 1 group.” Procedures in which more than one outflow tibial artery was achieved were classified as “runoff >1 group.”

**Figure 1:**
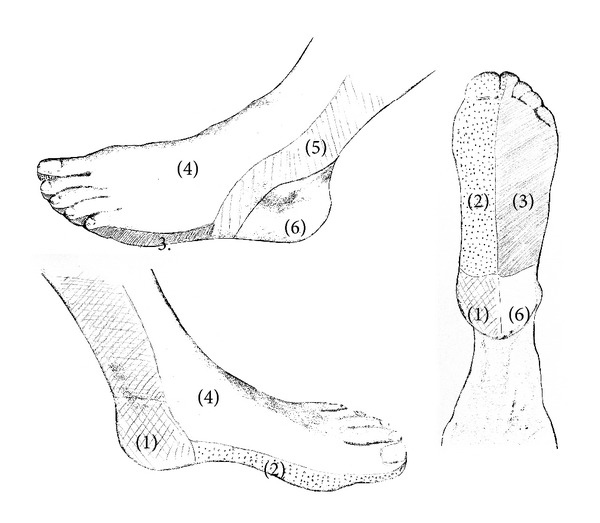
Angiosomes of the foot. Calcaneal branch (1); medial plantar branch (2); and lateral plantar branch (3) of the posterior tibial artery; dorsalis pedis angiosome (4); anterior branch (5) and calcaneal branch (6) of the peroneal artery.

Local perfusion of the ischemic ulcer obtained after revascularization was analyzed according to an angiosome model. Taylor and Palmer introduced this anatomical concept in 1987, widely used in modern plastic surgery, which is defined as 3-dimensional block of tissue supplied by a specific source artery and drained by a specific vein [14]. The foot can be divided into 6 angiosomes arising from the posterior tibial artery (*n* = 3), the anterior tibial artery (*n* = 1), and the peroneal artery (*n* = 2) [15, 16] (Figure 1). The posterior tibial artery gives rise to a calcaneal branch that supplies the medial ankle and plantar heel, a medial plantar branch that irrigates the medial plantar instep, and a lateral plantar branch that feeds the lateral forefoot, plantar midfoot, and entire plantar forefoot. The anterior tibial artery continues on to the dorsum of the foot as the dorsalis pedis. The peroneal artery gives rise to a calcaneal branch that supplies the plantar heel and lateral ankle and an anterior branch that feeds the anterior upper ankle. The flow provided by the tibial vessels is interconnected between angiosomes by arterial-arterial connections (collateral vessels). The pedal arch communicates the dorsal and plantar arterial flow, and the peroneal artery distal branches connect this vessel with the circulation from the anterior and posterior tibial arteries at the ankle and foot. On the other hand, foot toes and heal are “double secure” because each location could be supplied by two tibial arteries [15, 16]. Toes could be perfused through anterior tibial and posterior tibial artery branches. Heel could be supplied via posterior tibial artery and peroneal artery calcaneal branches. Therefore, digit ulcers that affected first and second toes were considered to be placed in dorsalis pedis angiosome and in medial plantar branch angiosome. Digit ulcers located in the rest of toes were considered to be placed in dorsalis pedis and lateral plantar branch angiosomes. Heel ulcers were considered to be located in both calcaneal branch of posterior tibial artery angiosome and calcaneal branch of peroneal artery angiosome.

The analysis according to the postoperative ischemic ulcer local perfusion was only performed in those procedures in which at least one patent tibial vessel was obtained. We excluded the “runoff 0” interventions in order to evaluate the influence of foot vessels patency on our cohort results. Preoperative and intraoperative angiographies were reviewed and procedures were classified using an angiosome anatomical model as ‘‘direct revascularization” (DR) if at least one feeding vessel supplied the injured angiosome and as ‘‘indirect revascularization” (IR) if it fed an unrelated angiosome. Indirect revascularization was subdivided into IR ‘‘through collaterals” (IRc) and ‘‘without collaterals” depending on the presence of collateral vessels to the affected angiosome (pedal arch and distal peroneal branches). The ulcer-angiosome and angiogram-angiosome assignment were done in a blinded fashion. Revascularization of digit ulcers by the ‘‘dorsalis pedis” or the plantar artery was considered as DR. The same exception was applied in heel ulcers perfused by the plantar artery or the calcaneal branch of the peroneal artery.

All patients were called for follow-up at 1 month, 3 months, and then every 6 months after the procedure was performed. Patency was assessed by ABI in all cases. Color duplex scanning was performed in those patients with noncompressible ABI, with stent implants or those who required complex endovascular revascularization procedures. Patients who showed clinical worsening or those with a drop in ABI >0.15 were initially assessed by color duplex scanning. Echographic restenosis was defined as the finding of a peak systolic velocity ratio >2.5 in the target lesion and only symptomatic restenosis were treated. Lower limbs angiography was performed prior to reintervention. Reinterventions were classified as major (conversion to bypass, thrombectomy, thrombolysis, or major surgical procedure) and minor (new endovascular procedure without the need for thrombectomy or thrombolysis).

The main objectives of the study were ischemic ulcer healing at 12 months and limb salvage at 24 months. Foot tissue lesions were considered as infected according to the CDC/NHSN surveillance definition of health care-associated infection criteria [17]. Local treatment included early debridement, abscess drainage, minor amputations, and wet dressings. Severe infections received broad-spectrum antibiotic therapy in accordance with our general protocol. The healing process was followed up at intervals of 1 to 2 weeks. All the written documentation and digital photographs recorded during follow-up were evaluated. We defined ‘‘healing time” as the time needed for the complete epithelialisation of the ischemic ulcer. ‘‘Major amputation” was defined as an amputation performed above the ankle. The ischemic ulcers that were not healed in the last follow-up visit of patients who subsequently died or required major amputation were never considered as healed.

The results of our cohort were also analyzed according to the definition of the different Objective Performance Goals proposed by the Society for Vascular Surgery for evaluating catheter-based treatment of CLI [18]. The incidence of acute myocardial infarction, stroke, or death from any cause (Major Adverse Cardiovascular Event (MACE)), the incidence of major amputation or major reintervention (Major Adverse Limb Event (MALE)), and the frequency of major amputation at 30 days were recorded as estimators of intervention safety.

Freedom from any MALE or perioperative death (freedom from MALE+POD), amputation-free survival, and overall survival at 24 months were calculated to discriminate between limb-specific results and survival-related results as CLI patients use to develop multiple adverse events that can greatly influence the revascularization outcomes and patients survival. Freedom from any reintervention or amputation (freedom from RAO) and freedom from restenosis, any reintervention or amputation (freedom from RAS), were measured as estimators of hemodynamic failure [18]. In this context, freedom from RAS was only calculated over 92 procedures because the data about untreated restenosis was not of enough quality in nine interventions due to incomplete hemodynamic follow-up information recorded.

All the previously mentioned endpoints were compared according to the number of tibial vessels attempted for revascularization, the number of tibial vessels finally achieved to the foot, and the local perfusion of the ischemic ulcer obtained after revascularization.

*Statistical Analysis*. Data was processed using the software packages SPSS 15.0 (Microsoft). Differences between groups were considered statistically significant for a *P* < 0.05 in 2-tailed test. The analysis of normality was carried out using the Kolmogorov-Smirnov and Shapiro-Wilk tests and continuous variables were compared using the Mann-Whitney *U*-test. Wilcoxon signed rank test was performed to measure differences in repeated measurements on a single continuous variable. The Chi-square and Fisher exact test were used to explore the differences between categorical variables. Ischemic ulcer healing at 12 months, limb salvage, and the different Objective Performance Goals-based endpoints at 24 months were measured by the Kaplan Meier method. Survival curves were compared using the Log-Rank test. Continuous variables are expressed as the median (interquartile range (p25–p75)) and the categoricals as percentages.

## 3. Results

One hundred and one primary infrapopliteal endovascular procedures performed in 92 diabetic patients with ischemic ulcers between January 1999 and December 2009 were retrospectively followed. All the revascularizated ischemic ulcers were limited to the foot. Most of the procedures were performed between 2005 and 2009 (74 (73%) endovascular techniques) and only 8 (8%) between 1999 and 2000. Median follow-up was 19 (9–38) months and 11 (10.8%) patients were lost. Demographic characteristics, comorbidities, ischemic ulcer description, and basal hemodynamic data are described in Table 1.

The TASC-II classification of the worst lesion treated was TASC-D in 79.2% surgeries. In 56 (55.4%) endovascular procedures the tibial intervention was combined with a femoro-popliteal angioplasty. Stents were used selectively in 9 (8.9%) procedures. Seven (6.8%) stents were implanted in the femoro-popliteal sector and 2 (1.9%) in the tibial sector. Multiple revascularization of the tibial sector was attempted in 52 (51.5%) interventions and single revascularization in 49 (48.5%). In 16 (15.8%) procedures a direct line of blood flow to the foot was not achieved (runoff 0 group). A single patent tibial vessel was obtained in 64 (63.4%) interventions (runoff 1 group) and more than one in 21 (20.8%) procedures (runoff >1 group). After excluding the runoff 0 group, DR of the ischemic ulcer was achieved in 46 (54.1%) procedures, IR “through collaterals” in 22 (25.9%) and IR “without collaterals” in 17 (20%) interventions (Table 2). Postoperative ankle-brachial index was significantly higher to the basal index recorded (0.54 (0.40–0.67) versus 0.84 (0.69–0.93) *P* < 0.001). Ankle-brachial index was noncompressible in 54 (53.5%) procedures.

### 3.1. Overall Results

The incidence of MACE, MALE, and major amputation at 30 days was 3%, 3%, and 2%, respectively. Ischemic ulcer healing at 12 months was 55.0% and limb salvage at 24 months was 74.9%. Freedom from MALE+POD at 24 months was 64.8%. We recorded an amputation-free survival rate of 63.3% after 2 years of follow-up. Overall survival at 24 months was 76.9%. Freedom from RAS and freedom from RAO at 24 months were 37.6% and 58.9%, respectively.

### 3.2. Results according to Number of Tibial Vessels Attempted for Revascularization

A multiple tibial revascularization attempt was more frequently performed in males and in patients with smoking history (Table 3). Combined treatment of the femoro-popliteal and tibial sector was more frequent in the single revascularization group. A direct line of blood flow to the foot was not achieved in 10 (20.4%) single revascularization attempts and in 6 (11.5%) multiple revascularization attempts (*P* = 0.22). More than one patent tibial vessel to foot was finally obtained in 18 (34.6%) procedures of the multiple revascularization group and in 3 (6.1%) interventions of the single revascularization group (*P* < 0.01) (Table 4).

#### 3.2.1. Safety Endpoints

The incidence of MACE (4.1% versus 1.9% *P* = 0.61), MALE (2.0% versus 3.8% *P* = 0.59), and major amputation at 30 days (0% versus 3.8% *P* = 0.49) was similar between single revascularization and multiple revascularization groups (Table 4).

#### 3.2.2. Ischemic Ulcer Healing and Limb Salvage

**Figure 2:**
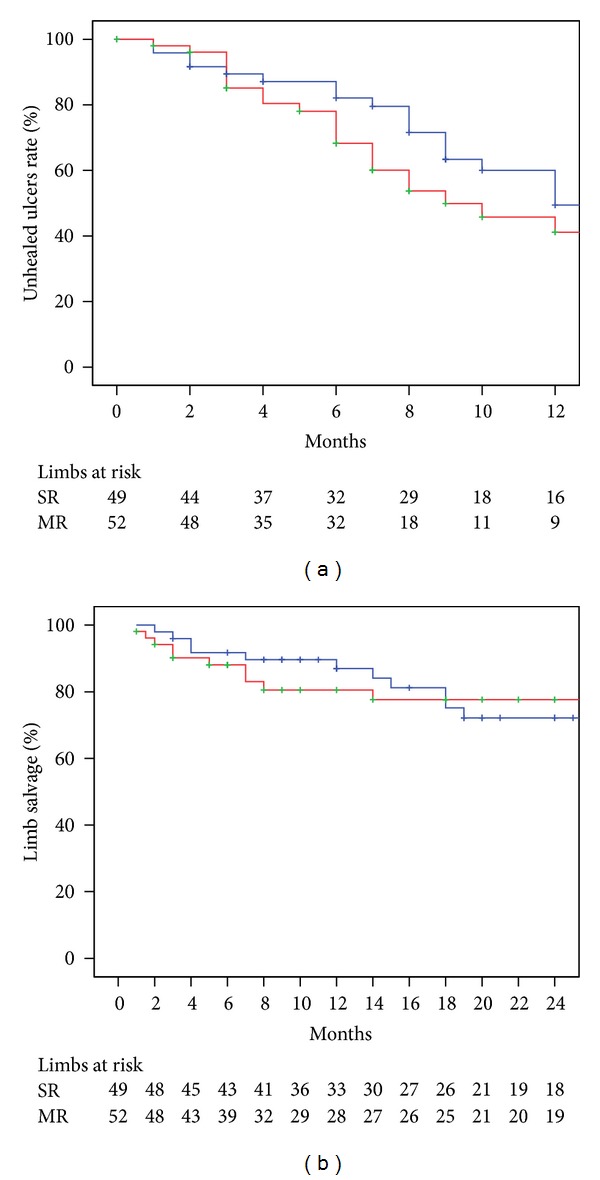
Ischemic ulcer healing at 12 months (a) and limb salvage at 24 months according to number of tibial vessels attempted for endovascular treatment. SR: single revascularization (Blue line); MR: multiple revascularization (red line). The standard error was <10% for the data shown.

Ischemic ulcer healing rate at 12 months was 50.6% in the single revascularization group and 58.8% in the multiple revascularization group (*P* = 0.18). Limb salvage at 24 months was similar between single revascularization and multiple revascularization groups (72.2% versus 77.6% *P* = 0.62) (Figure 2).

#### 3.2.3. Limb-Specific and Survival Related Results

Freedom from MALE-POD at 24 months was 62.1% in the single revascularization group and 71.3% in the multiple revascularization group (*P* = 0.33). There were no statistical differences in amputation-free survival at 24 months between single and multiple revascularization attempt strategy (62.4% versus 64.3% *P* = 0.96). Overall survival at 24 months was 78.7% in the single revascularization group and 75.2% in the multiple revascularization group (*P* = 0.99).

#### 3.2.4. Hemodynamic-Related Results

Freedom from RAS at 24 months was 24% in the single revascularization group and 43% in the multiple revascularization group (*P* = 0.35). There were no statistical differences between single and multiple revascularization attempt strategy in terms of freedom from RAO (50.5% versus 67.6% *P* = 0.25).

### 3.3. Results according to the Number of Patent Tibial Vessels Achieved to the Foot

Smoking history, ischemic heart disease, and previous strokes were more prevalent in runoff >1 group patients (Table 5). Postoperative ankle-brachial index was significantly lower in the runoff 0 group when it was compared with the values obtained after revascularization of the runoff 1 group (0.52 (0.57–0.66) versus 0.88 (0.77–0.93) *P* = 0.01) or the runoff >1 group (0.52 (0.57–0.66) versus 0.90 (0.77–0.97)). (Table 6).

#### 3.3.1. Safety Endpoints

The incidence of MACE, MALE, and major amputation at 30 days was similar between runoff 0 group, runoff 1 group, and runoff >1 group (Table 6).

#### 3.3.2. Ischemic Ulcer Healing and Limb Salvage

**Figure 3:**
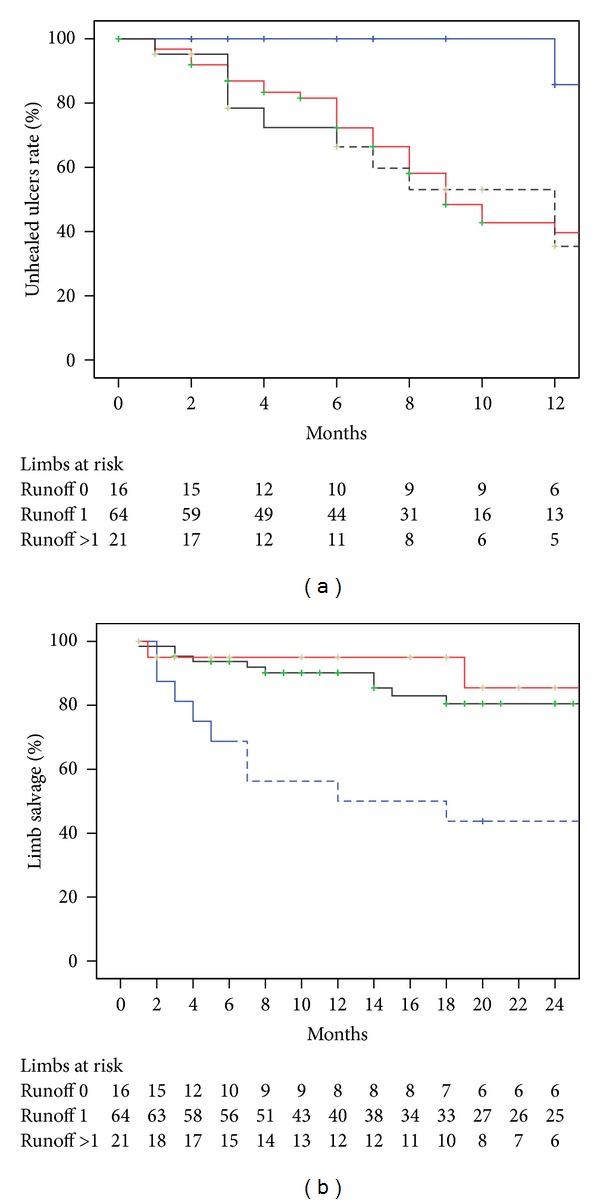
Ischemic ulcer healing at 12 months (a) and limb salvage at 24 months according to number of patent tibial vessels achieved to the foot. Runoff 0 = none patent tibial vessel achieved (blue line); Runoff 1 = one patent tibial vessel achieved (red line); Runoff >1 = more than one patent tibial vessel achieved (black line). The standard error was >10% for the data shown in dashed lines.

Ischemic ulcer healing rate at 12 months was much lower in the runoff 0 group when it was compared with healing rates of the runoff 1 group (14.3% versus 60.3% *P* = 0.01) or the runoff >1 group (14.3% versus 64.6% *P* = 0.003). There were no statistical differences in ulcer healing between runoff 1 group and runoff >1 group (60.3% versus 64.6% *P* = 0.43). Limb salvage at 24 months was lower in the runoff 0 group than in the runoff 1 group (43.8% versus 80.5% *P* = 0.001) or the runoff >1 group (43.8% versus 85.5% *P* = 0.006). There were no differences in terms of limb salvage at 24 months between runoff 1 group and runoff >1 group (80.5% versus 85.5% *P* = 0.50) (Figure 3).

#### 3.3.3. Limb-Specific and Survival Related Results

Freedom from MALE+POD at 24 months was lower in the runoff 0 group when it was compared to the runoff 1 group (25.0% versus 74.9% *P* > 0.001) or the runoff >1 group (25.0% versus 70.7% *P* = 0.006) and no differences were observed between runoff 1 group and runoff >1 group (74.9% versus 70.7% *P* = 0.62).

Amputation-free survival at 24 months was lower in runoff 0 group than in runoff 1 group (43.8% versus 64.3% *P* = 0.02) or runoff >1 group (43.8% versus 76.6% *P* = 0.04). There were no differences in terms of amputation-free survival at 24 months between runoff 1 group and runoff >1 group (64.3% versus 76.6% *P* = 0.50).

Overall survival at 24 months was 84.4% in runoff 0 group, 72.9% in runoff 1 group and 83.6% in runoff >1 group and no statistical differences were observed (runoff >1 versus runoff 1 *P* = 0.77; runoff >1 versus runoff 0 *P* = 0.74; and runoff 1 versus runoff 0 *P* = 0.74).

#### 3.3.4. Hemodynamic-Related Results

Freedom from RAS at 24 months was lower in the runoff 0 group when it was compared with runoff 1 group (18.8% versus 42.8% *P* = 0.01) and runoff >1 group (18.8% versus 42.9% *P* = 0.08). There were no differences between runoff 1 group and runoff >1 group (42.8% versus 42.9% *P* = 0.70).

Freedom from RAO at 24 months in the runoff 0 group was lower than the rates obtained in the runoff 1 group (25.0% versus 66.5% *P* > 0.001) and the runoff >1 group (25.0% versus 65.8% *P* = 0.02). There were no differences between runoff 1 group and runoff >1 group (66.5% versus 65.8% *P* = 0.50).

### 3.4. Results according to the Local Perfusion of the Ischemic Ulcer

Eighty-five procedures were analyzed after excluding 16 interventions in which no patent tibial artery to the foot was obtained. The results of these “runoff 0” interventions have been shown above.

Basal characteristics of the patients included are shown in Table 7. Combined treatment of the femoro-popliteal and the tibial sector was more frequently performed in the DR group when it was compared with the procedures in which IR “through collaterals” (43.5% versus 31.8% *P* = 0.06) or IR “without collaterals” (43.5% versus 17.6% *P* = 0.009) was obtained. A multiple tibial revascularization attempt was more frequently performed in those procedures in which a DR was obtained than in those in which an IR “through collaterals,” (67.4% versus 36.4% *P* = 0.01) or an IR “without collaterals” (67.4% versus 41.2% *P* = 0.06) was achieved. DR of the ischemic ulcer was more frequent in procedures that obtained more than one patent tibial vessel and IR “through collaterals” was more frequent in those that achieved only a single patent tibial artery to the foot. Postoperative ankle-brachial index was similar between DR, IR “through collaterals” and IR “without collaterals” groups as it is expected because at least one patent tibial artery to the foot was obtained in all the analyzed groups (Table 8).

#### 3.4.1. Safety Endpoints

The incidence of MACE, MALE, and major amputation at 30 days was similar between DR, IR “through collaterals,” and IR “without collaterals” groups (Table 8).

#### 3.4.2. Ischemic Ulcer Healing and Limb Salvage

**Figure 4:**
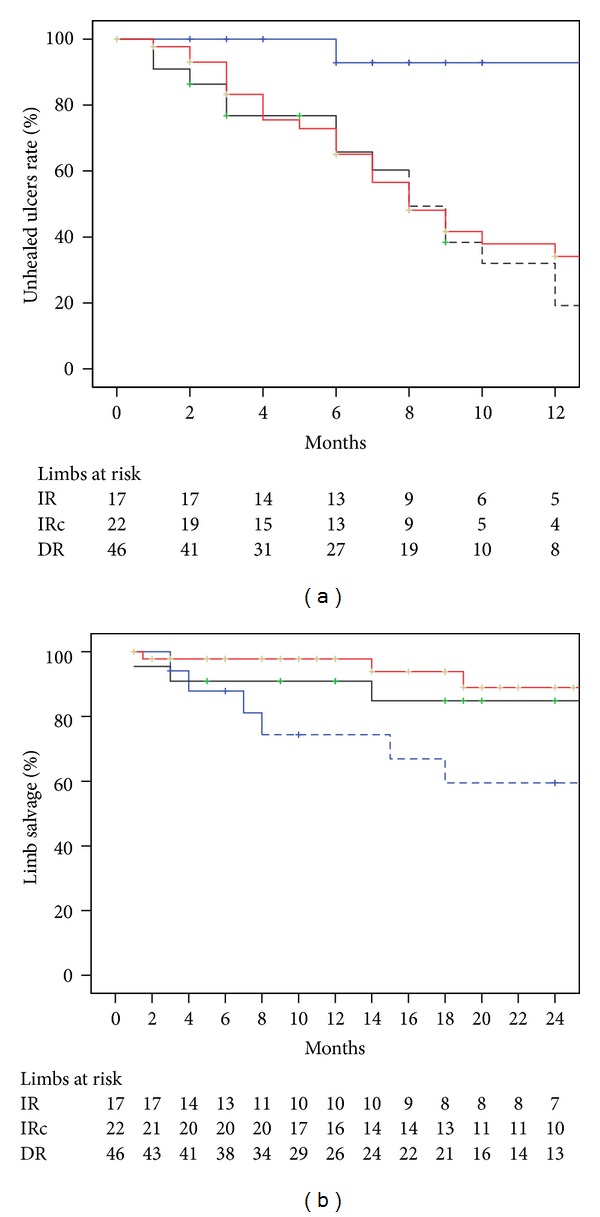
Ischemic ulcer healing at 12 months (a) and limb salvage at 24 months according to an angiosome classification of the ulcers. IR: indirect revascularization without collaterals (blue line); IRc: indirect revascularization through collateral vessels (black line); DR: direct revascularization (red line). The standard error was >10% for the data shown in dashed lines.

Ischemic ulcer healing at 12 months was lower in IR “without collaterals” group when it was compared with DR (7.1% versus 66.0% *P* = 0.001) or IR “through collaterals” groups (7.1% versus 68.0% *P* < 0.001). Ischemic ulcer healing rate at 12 months was similar between DR and IR “through collaterals” groups (66.0% versus 68.0% *P* = 0.38). Limb salvage at 24 months was lower in the procedures in which an IR “without collaterals” of the ischemic ulcer was achieved when it was compared with DR (59.0% versus 88.9% *P* = 0.04) or IR “through collaterals” groups (59.0% versus 84.8% *P* = 0.06). Limb salvage at 24 months was similar between DR and IR “through collaterals” groups (88.9% versus 84.8% *P* = 0.45) (Figure 4).

#### 3.4.3. Limb-Specific and Survival-Related Results

Freedom from MALE+POD at 24 months was lower in IR “without collaterals” group when it was compared with DR (45.9% versus 80.9% *P* = 0.005) or IR “through collaterals” groups (45.9% versus 80.6% *P* = 0.06). There were no differences between DR and IR “through collaterals” groups (80.9% versus 80.6% *P* = 0.38).

Amputation-free survival at 24 months was 67.5% in the DR group, 73.3% in the IR “through collaterals” group, and 61.9% in IR “without collaterals” group, and no statistical differences were observed (DR versus IR “through collaterals” *P* = 0.29; DR versus IR “without collaterals” *P* = 0.31; IR “through collaterals” versus IR “without collaterals” *P* = 0.10). Overall survival at 24 months was 73.6% in the DR group, 76.1% in the IR “through collaterals” group and 84.8% in the IR “without collaterals” group (DR versus IR “through collaterals” *P* = 0.24; DR versus IR “without collaterals” *P* = 0.71; IR “through collaterals” versus IR “without collaterals” *P* = 0.57).

#### 3.4.4. Hemodynamic-Related Results

There was a tendency towards a lower freedom from RAS rate at 24 months in the IR “without collaterals” group when it was compared with DR group (22.5% versus 53.0% *P* = 0.08). No differences in terms of freedom from RAS were observed between DR and IR “through collaterals”(53.0% versus 43.9% *P* = 0.19) and between IR “through collaterals” and IR “without collaterals” (43.9% versus 22.5% *P* = 0.66).

Freedom from RAO at 24 months was lower in the IR “without collaterals” group than in DR group (38.5% versus 74.0% *P* = 0.02). There was a tendency towards a lower freedom from RAS rate in the IR “without collaterals” group when it was compared with IR “through collaterals” group (38.5% versus 77% *P* = 0.08). No differences were observed between DR and IR “through collaterals” groups (74.0% versus 77.0% *P* = 0.78).

## 4. Discussion

The care of reperfused ischemic ulcers is a major postoperative problem due to the slow tissue healing time and the frequent need for associated techniques on the ischemic wound. Delayed healing is especially important in patients with diabetes mellitus. This disease produces a number of complex biomechanical, neuropathogenic, and immunogenic foot disorders, which are able to reduce the capacity of tissue regeneration [19]. In our experience, diabetic patients are more than twice at risk of developing healing failure than nondiabetic patients [12] and only 55% of the ulcers in our cohort of diabetics were healed after 1 year of follow-up. In this context, obtaining a direct line of blood flow to the foot is mandatory to fulfil the nutritive requirements needed for the ulcer healing process [5–9]. In our cohort, the group of patients in whom none patent tibial vessel to the foot was obtained during the endovascular procedure was more prone to develop delayed wound healing and needed major amputation more frequently. In fact, this patients also presented a higher rate of major adverse limb events due to the higher incidence of limb loss and major reinterventions, that were, mostly, distal bypass conversions to tibial outflow vessels inaccessible to an endovascular approach.

One of the main advantages of the endovascular treatment is the possibility to “navigate” through the patient's infragenicular vascular anatomy. Endoluminal techniques offer the chance of treating more than one tibial vessel and this strategy could be associated with better clinical outcomes due to a better hypothetical hemodynamic improvement. According to Faglia et al., the risk of major amputation could increase up to eight times for each obstructed crural artery with alarming proportions in those procedures in which all three crural arteries were occluded [6]. However, other authors have observed that clinical results of revascularization are subject to blood flow restoration to the ischemic area rather than the number of tibial runoff vessels achieved to the foot. Iida et al. pointed out that higher limb salvage rates, observed in their angiosome-oriented endovascular group, were independent of the number of runoff vessels [13]. In our experience, those procedures in which more than one patent tibial artery was obtained (runoff >1) achieved similar ankle-brachial index improvement than those in which a single outflow (runoff 1) vessel was reperfused. Furthermore, the runoff >1 group did not show any clinical or hemodynamic improvement during follow-up when it was compared with runoff 1 group. In addition, in our cohort a multiple tibial endovascular revascularization attempt finally achieved more than one patent vessel to the foot in only 34% of the cases. Clinical follow-up results were similar between multiple revascularization and single revascularization attempt. In this sense, the infrainguinal diffuse vascular lesions seen in diabetic patients with CLI could hamper this specific endovascular revascularization strategy. In one report that used limb angiography to study 417 diabetic CLI patients with pedal tissue loss, 74% of the vascular lesions were below the knee, 66% were occlusive lesions, and all the tibial vessels were occluded in 28% of the patients [4]. Diabetic patients with infrapopliteal atherosclerosis frequently develop concentric continuous vascular wall calcifications, situation that could also technically limit the effectiveness of the endovascular treatment option [3]. Therefore, according to these data, we argue that obtaining a single patent tibial artery to the foot is enough to achieve good clinical results not only in bypass arena but also in an endovascular strategy of foot reperfusion. However, a multiple revascularization attempt seems secure at 30 days and could be used in selected cases.

On the other hand, the success of revascularization, although essential to ensure limb salvage, does not completely reduce the risk of delayed ischemic ulcer healing and major amputation. A healing failure rate and limb loss of around 15% have been reported in patients with pedal tissue loss and successful tibial vein bypasses [5, 20]. Systemic factors associated with delayed healing, ulcer characteristics, wound anatomical location, patients functional status, and the inadequate postoperative wound local treatment could explain part of the clinical failure despite successful arterial reconstruction [12, 21–23]. However, ischemic ulcers could also fail to heal because inadequate connections between the revascularizated tibial artery and the local ischemic area. The angiosome anatomical concept could provide the theoretical basis for the analysis of this potential cause of delayed healing. In fact, this anatomical model has been recently applied to the revascularization planning of CLI patients with encouraging results. Several reports have shown that obtaining direct arterial blood flow through the tibial artery that specifically feeds the injured pedal angiosome could lead to better outcomes in terms of ischemic ulcer healing and limb salvage [7–9, 13]. Alexandrescu et al. reported a limb salvage rate of 84% and ischemic ulcer healing rate of 73% at 36 months in diabetic patients with tissue loss, who underwent endovascular lower limb procedures in which specific revascularization of the injured angiosome was considered [7]. Iida et al. recently observed limb preservation in 86% of the angiosome-oriented group versus 69% in the nonspecific group, in 203 consecutive limbs with ischemic ulcers undergoing endovascular revascularization [13]. However, as stated previously, CLI diabetic patients often develop a multilevel occlusive arterial disease that prevents direct revascularization of the injured angiosome [3, 4, 9]. In our cohort, 45% of the endovascular procedures in which at least one patent tibial artery was obtained to the foot did not achieve a direct revascularization of the ulcer. In this group of individuals, foot collateral reserve represents the only potential blood flow route for ischemic ulcer healing. Foot angiosomes are interconnected by a small sized collateral network called choke-vessels system that could be able to reperfuse the ischemic tissue mainly in healthy individuals. Pedal angiosomes are also linked through medium and large sized collateral vessels, called arterial-arterial connections like the pedal arch and peroneal distal branches [16]. The skin microcirculatory impairment related to endothelial dysfunction altered hemorheology and the autonomic denervation seen in CLI diabetic patients could lead to foot choke collateral network depletion in this individuals [1, 3]. Furthermore, the aggressive form of atherosclerosis observed in diabetics could also affect arterial-arterial connections between angiosomes [1, 3]; however this collateral vessels could be useful in distal arterial reconstruction planning when patent. In fact, in this study we have observed that indirect revascularization of the ulcer through arterial-arterial connections (IR “through collaterals”) provided similar results in terms of ischemic ulcer healing at 12 months and limb salvage at 24 months than the direct revascularization of the pedal tissue defect. These data are congruent with the observed results of a previous report in which a cohort of endovascular and surgical infrapopliteal procedures was analyzed [9]. Limb-specific, survival-related, and hemodynamic-related outcomes were also similar between the DR group and the IR “through collaterals” group. Nevertheless, those patients in whom blood flow was directed to the ulcer through the choke collateral network (IR “without collaterals”), impaired in diabetic patients, achieved a total epithelization of the ulcer in only 7% of the cases at 12 months, situation that lead to major amputation in a great number of these subjects. These data could explain in part why mayor adverse limb events occurred more frequently in the IR “without collaterals” group. These patients were also more prone to require a distal vein bypass conversion to a more suitable tibial vessel because of ulcer delayed healing.

Therefore, according to these results, the depleted choke collateral network seems not to be able to successfully reperfuse foot ischemic wounds in CLI diabetic patients. However, blood flow restoration to the ulcer through patent arterial-arterial connections like pedal arch or distal peroneal branches provided similar results to those obtained after reperfusion of the ulcer through its specific tibial artery in our cohort. The influence of these collateral vessels on distal revascularization results has been widely discussed with contradictory conclusions. Berceli et al. in a retrospective cohort of pedal distal bypasses performed for heel ulcerations described a tendency towards a higher ulcer healing rate in those with a patent pedal arch though no statistical differences were recorded [5]. In the endovascular field, Kawarada et al. observed better wound healing results in a cohort of 106 ulcerated limbs that underwent to successful stent-assisted infrapopliteal angioplasties if angiographic pedal arch patency was confirmed [24]. On the other hand, the results of distal revascularization based on the peroneal artery have been reported to be inferior to those obtained after the revascularization of the anterior or the posterior tibial artery. In a retrospective series of 420 consecutive diabetic patients, Faglia et al. observed that the reperfusion through the peroneal artery alone is not sufficient to avoid major amputation in some patients, but author did not analyze the peroneal distal branches patency [6]. However, other authors have found similar clinical results between peroneal artery and other tibial arteries after endovascular [25] or surgical bypass procedures [26]. In this sense, we argue that the disparity of the results between publications that have focused on foot outflow vessels could be explained in part by the lack of a foot anatomical ulcer classification and the absence of data about foot and ankle collateral patency in some studies. Angiosome classification of the foot tissue loss could provide the theoretical basis to explore the influence of distal collateral vessels on distal revascularization ischemic ulcer healing and limb salvage.

This study has some limitations. It is a retrospective analysis; therefore the groups compared are not homologous, as they were not randomly assigned, situation that prevents from firm conclusions. We could not assess the size of the wounds and the different local treatment applied to the ischemic ulcer. Toe pressure was not routinely measured in those patients with noncompressible ABI. Therefore, hemodynamic data should be interpreted with caution. Most of the procedures were performed during the last 5 years of follow-up. This imbalance could be explained by our learning curve and the progressive technical improvement that experience the endovascular techniques in last years making this type of treatment more accessible for patients with CLI. As a result, the procedures indication may be varied over the years as we covered a wide enrollment period. The number of cases of some of the analyzed groups was small due to the limited sample size obtained following our inclusion criteria. This limitation explains why standard errors of some of the Kaplan-Meier curves exceeded the 10% standard error.

## 5. Conclusions

With these limitations in mind, our results suggest that obtaining a direct line of blood flow to the foot during a infrapopliteal endovascular procedure performed in diabetics for CLI and tissue loss is mandatory to achieve ischemic ulcer healing and limb salvage. A multiple endovascular revascularization attempt seems secure, although this strategy does not improve the revascularization results. Clinical outcomes are subject to the restoration of blood flow to the local ischemic and are independent of the number of patent tibial vessels obtained after revascularization. In this context, the restoration of blood flow to the ischemic ulcer through a medium or large size collateral vessel could provide similar results in terms of ulcer healing and limb salvage rates to those obtained via its specific source tibial artery. Therefore, a careful study of the foot collateral vessels anatomy should be done before an endovascular infrapopliteal procedure is performed.

According to the data draw in this study we suggest a distal endovascular planning in three steps. First, it is mandatory to obtain a direct straight line to the foot through the easier-to-treat tibial artery, even if the injured angiosome is not anatomically fed because this circumstance improves the results of the revascularization significantly. Second, if in the first step we did not achieve blood flow to the ulcer it is advisable to attempt the revascularization of another tibial vessel specifically related to the ulcer or indirectly-related through collateral vessels. Third, if it is not possible to obtain a direct line to the foot, a distal vein bypass has to be considered if feasible.

## Figures and Tables

**Table 1:** Basal characteristics of the subjects included.

Basal characteristics	*n* = 101
Age (years)	72 (64–77)
Males	62 (61.4%)
Smoking history	75 (74.3%)
Dyslipidemia	33 (32.7%)
Hypertension	73 (72.3%)
Ischemic heart disease	30 (29.7%)
Chronic obstructive pulmonary disease	6 (5.9%)
Stroke	20 (19.8%)
Preoperative ABI*	0.54 (0.40–0.67)
Noncompressible ABI*	54 (53.5%)
Infected ulcers	37 (36.6%)
Injured angiosome	
Dorsalis pedis angiosome	79 (78.2%)
Medial plantar branch angiosome	54 (53.5%)
Lateral plantar branch angiosome	26 (25.7%)
Posterior tibial a. calcaneal angiosome	11 (10.9%)
Peroneal a. calcaneal angiosome	9 (8.9%)
Peroneal a. anterior angiosome	2 (2.0%)

*ABI: ankle-brachial index.

**Table 2:** TASC-II classification for the worst lesion treated and perioperative data of the subjects included.

Perioperative data	*n* = 101
TASC-B	6 (5.9%)
TASC-C	15 (14.9%)
TASC-D	80 (79.2%)
Combined treatment*	56 (55.4%)
Debridement	11 (10.9%)
Minor amputation	28 (27.7%)
Multiple revascularization	52 (51.5%)
Runoff 0	16 (15.8%)
Runoff 1	64 (63.4%)
Runoff > 1	21 (20.8%)
DR^#^	46 (54.1%)
IR^†^ “through collaterals”	22 (25.9%)
IR^†^ “without collaterals”	17 (20.0%)
Postoperative ABI^&^	0.84 (0.69–0.93)
MACE^+^ at 30 days	3 (3.0%)
MALE^*μ*^ at 30 days	3 (3.0%)
Major amputation at 30 days	2 (2.0%)

*Combined treatment: combined treatment of the femoropopliteal and the infrapopliteal sector; ^#^DR: direct revascularization; ^†^IR: indirect revascularization; ^&^ABI: ankle-brachial index; ^+^MACE: major adverse cardiovascular event; ^*μ*^MALE: major adverse limb event.

**Table 3:** Basal characteristics according to the number of tibial vessels attempted for revascularization.

Basal characteristics	SR^#^ (*n* = 49)	MR^†^ (*n* = 52)	*P* value
Age (years)	72 (66–80)	72 (63–76)	0.39
Males	24 (48.9%)	38 (73.0%)	**0.01**
Smoking history	30 (61.2%)	45 (86.5%)	**0.04**
Dyslipidemia	18 (36.7%)	15 (28.8%)	0.39
Hypertension	36 (73.5%)	37 (71.2%)	0.79
Ischemic heart disease	15 (30.6%)	15 (28.8%)	0.84
Chronic obstructive pulmonary disease	3 (6.1%)	3 (5.8%)	0.94
Stroke	10 (10.4%)	10 (19.2%)	0.88
Preoperative ABI*	0.54 (0.44–0.68)	0.54 (0.35–0.67)	0.55
Noncompressible ABI*	24 (49.0%)	30 (57.7%)	0.38
Infected ulcers	17 (34.7%)	20 (38.5%)	0.69
Injured angiosome			
Dorsalis pedis angiosome	40 (81.6%)	39 (75.0%)	0.42
Medial plantar branch angiosome	25 (51.0%)	29 (55.8%)	0.63
Lateral plantar branch angiosome	15 (30.6%)	11 (21.2%)	0.27
Posterior tibial a. calcaneal angiosome	6 (12.2%)	5 (9.6%)	0.67
Peroneal a. calcaneal angiosome	4 (8.2%)	5 (9.6%)	0.53
Peroneal a. anterior angiosome	0 (0%)	2 (2.0%)	0.49

*ABI: ankle-brachial index; ^#^SR: single revascularization; ^†^MR: multiple revascularization.

**Table 4:** TASC-II classification for the worst lesion treated and perioperative data according to the number of tibial vessels attempted for revascularization.

Perioperative data	SR^+^ (*n* = 49)	MR^*μ*^ (*n* = 52)	*P* value
TASC-B	4 (8.2%)	2 (3.8%)	0.35
TASC-C	7 (14.3%)	8 (15.4%)	0.87
TASC-D	38 (77.6%)	42 (80.8%)	0.69
Combined treatment*	32 (65.3%)	24 (46.2%)	**0.05**
Debridement	6 (12.2%)	5 (9.6%)	0.67
Minor amputation	14 (28.6%)	14 (26.9%)	0.85
Runoff 0	10 (20.4%)	6 (11.5%)	0.22
Runoff 1	36 (73.5%)	28 (53.8%)	**0.04**
Runoff > 1	3 (6.1%)	18 (34.6%)	**<0.001**
Postoperative ABI^#^	0.90 (0.67–0.94)	0.83 (0.68–0.92)	0.86
MACE^†^ at 30 days	2 (4.1%)	1 (1.9%)	0.61
MALE^&^ at 30 days	1 (2.0%)	2 (3.8%)	0.59
Major amputation at 30 days	0 (0%)	2 (3.8%)	0.49

*Combined treatment: combined treatment of the femoropopliteal and the infrapopliteal sector; ^#^ABI: ankle-brachial index; ^†^MACE: major adverse cardiovascular events; ^&^MALE: major adverse limb events; ^+^SR: single revascularization; ^*μ*^MR: multiple revascularization.

**Table 5:** Basal characteristics according to the patent tibial vessels achieved to the foot.

Basal characteristics				*P* value		
	Runoff 0 (*n* = 16)	Runoff 1 (*n* = 64)	Runoff > 1 (*n* = 21)	Runoff > 1 versus runoff 1	Runoff > 1 versus runoff 0	Runoff 1 versus runoff 0
Age (years)	74 (59–79)	72 (64–77)	70 (63–75)	0.46	0.57	0.87
Males	12 (75.0%)	35 (54.7%)	15 (71.4%)	0.17	0.80	0.14
Smoking history	13 (81.3%)	43 (67.2%)	19 (90.5%)	**0.03**	0.41	0.36
Dyslipidemia	7 (43.8%)	19 (29.7%)	7 (33.3%)	0.75	0.51	0.28
Hypertension	10 (62.5%)	48 (75.0%)	15 (71.4%)	0.74	0.56	0.31
Ischemic heart disease	4 (25.0%)	16 (25.0%)	10 (47.6%)	**0.05**	0.16	0.19
Chronic obstructive pulmonary disease	0 (0%)	6 (9.4%)	0 (0%)	0.32	n.a	0.34
Stroke	2 (12.5%)	10 (15.6%)	8 (38.1%)	**0.02**	**0.08**	0.55
Preoperative ABI*	0.46 (0.35–0.56)	0.55 (0.43–0.68)	0.61 (0.37–0.65)	0.55	0.28	0.21
Noncompressible ABI*	10 (62.5%)	31 (48.4%)	13 (61.9%)	0.28	0.97	0.31
Infected ulcers	5 (31.3%)	24 (37.5%)	8 (38.1%)	0.96	0.66	0.64
Injured angiosome						
Dorsalis pedis angiosome	14 (87.5%)	51 (79.7%)	14 (66.7%)	0.22	0.24	0.72
Medial plantar branch angiosome	9 (56.3%)	32 (50.0%)	13 (61.9%)	0.34	0.72	0.65
Lateral plantar branch angiosome	4 (25.0%)	18 (28.1%)	4 (19.0%)	0.56	0.70	0.53
Posterior tibial a. calcaneal angiosome	1 (6.3%)	6 (9.4%)	4 (19.0%)	0.25	0.36	0.57
Peroneal a. calcaneal angiosome	1 (6.3%)	4 (6.3%)	4 (19.0%)	0.09	0.36	0.68
Peroneal a. anterior angiosome	0 (0%)	2 (3.1%)	0 (0%)	0.56	n.a	0.63

*ABI: ankle-brachial index.

**Table 6:** TASC-II classification for the worst lesion treated and perioperative data according to the patent tibial vessels achieved to the foot.

Perioperative data				*P* value		
	Runoff 0 (*n* = 16)	Runoff 1 (*n* = 64)	Runoff > 1 (*n* = 21)	Runoff > 1 versus runoff 1	Runoff > 1 versus runoff 0	Runoff 1 versus runoff 0
TASC-B	0 (0%)	5 (7.8%)	1 (4.8%)	0.63	0.37	0.57
TASC-C	4 (25.0%)	9 (14.1%)	2 (9.5%)	0.72	0.20	0.28
TASC-D	12 (75.0%)	50 (78.1%)	18 (85.7%)	0.54	0.43	0.74
Combined treatment*	9 (56.3%)	25 (39.1%)	11 (52.4%)	0.28	0.81	0.21
Multiple revascularization	6 (11.5%)	28 (53.8%)	18 (34.6%)	**0.001**	**0.005**	0.65
Debridement	3 (18.8%)	6 (9.4%)	2 (9.5%)	0.98	0.63	0.37
Minor amputation	6 (37.5%)	19 (29.7%)	3 (14.3%)	0.25	0.13	0.54
Postoperative ABI^#^	0.52 (0.57–0.66)	0.88 (0.73–0.93)	0.90 (0.77–0.97)	0.54	**0.01**	**0.01**
MACE^†^ at 30 days	0 (0%)	3 (4.7%)	0 (0%)	0.57	n.a	0.50
MALE^&^ at 30 days	1 (4.8%)	2 (3.1%)	0 (0%)	0.57	0.56	0.63
Major amputation at 30 days	1 (4.8%)	1 (1.6%)	0 (0%)	0.43	0.56	0.80

*Combined treatment: combined treatment of the femoropopliteal and the infrapopliteal sector; ^#^ABI: ankle-brachial index; ^†^MACE: major adverse cardiovascular event; ^&^MALE: major adverse limb event.

**Table 7:** Basal characteristics according to the local perfusion of the ischemic ulcer.

Basal characteristics				*P* value		
	DR^#^ (*n* = 46)	IR^†^ “through collaterals” (*n* = 22)	IR^†^“without collaterals” (*n* = 17)	DR^#^ versus IR^†^ “through collaterals”	DR^#^ versus IR^†^ “without collaterals”	IR^#^ “through collaterals versus IR^†^ “without collaterals”
Age (years)	72 (63–78)	72 (68–75)	69 (63–77)	0.94	0.99	0.60
Males	30 (65.2%)	11 (50.0%)	9 (52.9%)	0.23	0.37	0.85
Smoking history	36 (78.3%)	15 (68.2%)	11 (64.7%)	0.36	0.27	0.81
Dyslipidemia	13 (28.3%)	9 (40.9%)	4 (23.5%)	0.29	0.70	0.25
Hypertension	31 (67.4%)	18 (81.8%)	14 (82.4%)	0.26	0.35	0.96
Ischemic heart disease	17 (36.9%)	5 (22.7%)	4 (23.5%)	0.24	0.31	0.95
Chronic obstructive pulmonary disease	3 (6.5%)	2 (9.1%)	1 (5.9%)	0.65	0.92	0.70
Stroke	9 (19.6%)	6 (27.3%)	3 (17.3%)	0.47	0.86	0.70
Preoperative ABI*	0.50 (0.41–0.68)	0.60 (0.48–0.74)	0.64 (0.32–0.67)	0.30	0.79	0.48
Noncompressible ABI*	25 (54.3%)	9 (40.9%)	7 (41.2%)	0.30	0.75	0.26
Infected ulcers	6 (35.3%)	8 (36.4%)	18 (39.1%)	0.82	0.78	0.94
Injured angiosome						
Dorsalis pedis angiosome	37 (80.4%)	16 (72.7%)	12 (70.6%)	0.47	0.40	0.88
Medial plantar branch angiosome	24 (52.2%)	11 (50.0%)	10 (58.8%)	0.86	0.63	0.58
Lateral plantar branch angiosome	10 (21.7%)	9 (40.9%)	3 (17.6%)	0.09	0.51	0.16
Posterior tibial a. calcaneal angiosome	5 (10.9%)	2 (9.1%)	3 (17.6%)	0.59	0.67	0.63
Peroneal a. calcaneal angiosome	5 (10.9%)	1 (4.5%)	2 (11.8%)	0.65	0.61	0.57
Peroneal a. anterior angiosome	2 (4.3%)	0 (0%)	0 (0%)	0.45	0.53	n.a

*ABI: ankle-brachial index; ^#^DR: direct revascularization; ^†^IR: indirect revascularization.

**Table 8:** TASC-II classification for the worst lesion treated and perioperative data according to the local perfusion of the ischemic ulcer.

Perioperative data				*P* value		
	DR^+^ (*n* = 46)	IR^*μ*^ “through collaterals” (*n* = 22)	IR^*μ*^ “without collaterals” (*n* = 17)	DR^+^ versus IR “through collaterals”	DR^+^ versus IR^*µ*^ “without collaterals”	IR^*μ*^ “through collaterals versus IR^*μ*^ “without collaterals”
TASC-B	3 (6.5%)	3 (13.6%)	0 (0%)	0.38	0.55	0.24
TASC-C	4 (8.7%)	3 (13.6%)	4 (23.5%)	0.67	0.19	0.67
TASC-D	39 (84.8%)	16 (72.7%)	13 (76.5%)	0.23	0.46	0.79
Combined treatment*	20 (43.5%)	7 (31.8%)	3 (17.6%)	**0.06**	**0.009**	0.46
Debridement	4 (8.7%)	2 (9.1%)	2 (11.8%)	0.95	0.65	0.78
Minor amputation	12 (26.1%)	7 (31.8%)	3 (17.6%)	0.62	0.74	0.46
Multiple revascularization	31 (67.4%)	8 (36.4%)	7 (41.2%)	**0.01**	**0.06**	0.75
Runoff 1	28 (60.9%)	20 (90.9%)	16 (94.1%)	**0.01**	**0.01**	0.70
Runoff > 1	18 (39.1%)	2 (9.1%)	1 (5.9%)	**0.01**	**0.01**	0.70
Postoperative ABI^#^	0.85 (0.70–0.95)	0.91 (0.82–0.95)	0.80 (0.65–0.91)	0.30	0.79	0.48
MACE^†^ at 30 days	1 (2.2%)	2 (9.1%)	0 (0%)	0.24	0.54	0.49
MALE^&^ at 30 days	1 (2.2%)	1 (4.5%)	1 (5.9%)	0.54	0.47	0.85
Major amputation at 30 days	1 (2.2%)	1 (4.5%)	0 (0%)	0.54	0.73	0.37

*Combined treatment: combined treatment of the femoropopliteal and the infrapopliteal sector; ^#^ABI: ankle-brachial index; ^†^MACE: major adverse cardiovascular event; ^&^MALE: major adverse limb event; ^+^DR: direct revascularization; ^*μ*^IR: indirect revascularization.
